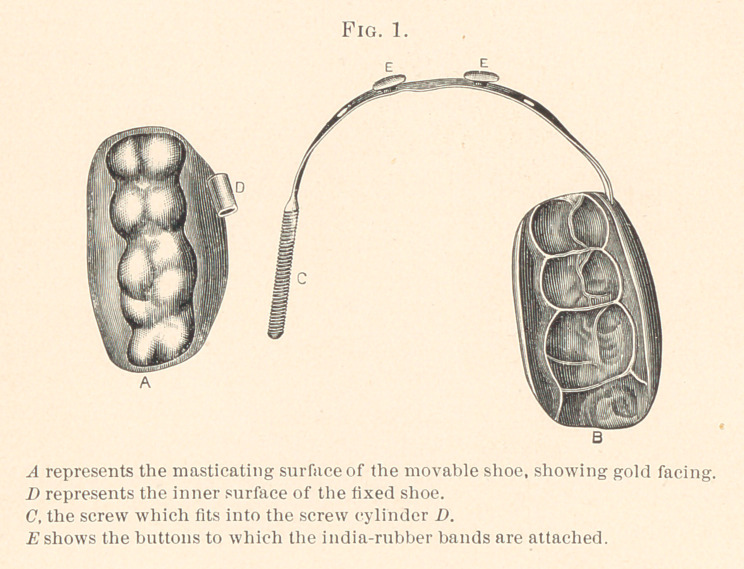# Odontological Society of Pennsylvania

**Published:** 1893-07

**Authors:** 


### ODONTOLOGICAL SOCIETY OF PENNSYLVANIA.

The regular meeting was held at 1228 Walnut Street, Philadelphia, May 13, 1893.

The subject for discussion was "Corrective Dentistry: Its Present Status."

The President, Dr. Louis Jack, called upon Dr. Guilford.

Dr. Guilford opened his remarks by stating that Dr. Bonwill could not have read his work on orthodontia carefully, for if he had he would have noticed a description and illustration of the appliance described by Dr. Bonwill, and properly credited to Dr. H. A. Baker, of Boston.

Continuing, Dr. Guilford said, "Appliances for the correction of irregularity are so varied in character and great in number that we can only take time to speak of them in a general way.

"In the earlier days force was principally derived from the elasticity of metals in the form of bars and springs, sometimes assisted incidentally with ligatures and wooden wedges; but after the introduction of caoutchouc, rings cut from rubber tubing and vulcanite plates were added to our list of materials available for exerting power. The latter, on account of their great adaptability and ease of construction, for many years largely superseded the use of metals, but the tendency nowadays seems to be to return to the use of metallic appliances, not only on account of their greater power, but because of their directness of action and their occupancy of less space in the mouth. In their construction the more expensive metals, such as gold, platinum, and their combinations, were formerly used; but with the advent of steel in the form of piano-wire and the introduction of German silver, it was found that equally efficient appliances could be constructed and at less cost. For this reason they have come into very general use. The chief objection to their employment is their oxidability, which soon renders them unsightly and seriously interferes with the movement of parts, such as nuts upon bolts and rods in tubes. To avoid this I was led to use platinized silver, which does not tarnish like German silver, and which, unlike steel, can be joined with hard solder without injuring its properties.

"It is an alloy composed of one part of platinum to two of silver, and was introduced from England, where for many years it has been used in the form of wire posts for the support of tube-teeth and in sheet form as a base for partial dentures. Its elasticity is little less than that of platinized gold, while its cost is about one-third that of gold.

"Its many valuable properties render it a most useful and serviceable material, and I now use it almost to the exclusion of other metals in the construction of all parts of regulating appliances.

"Rubber or vulcanite plates serve an admirable purpose in expanding the arch and in producing many simple movements of teeth, but the necessity for removing them at times for cleansing often tempts the patient to remove them when they should not be removed, and thus our results are frequently delayed and sometimes greatly interfered with. It is best not to confine one's self to any single system or method in regulating, but to use such parts of any method or to devise such appliance as seems best adapted for the case in hand.

"In regard to retaining devices, I think that in nearly all cases they should be of such character as to be non-removable, for in removing and replacing them the teeth are more or less disturbed, and this hinders their growing firm in their new positions. Retaining fixtures serve their purpose best when cemented to the teeth and left there for a period of not less than six months. Appliances attached to teeth by means of bands or clasps will after a long time cause injury to the teeth, but when cemented in place no injury will result."

*Dr. Darby.*—I think since the year 1871, when I heard Dr. Walter Coffin, of London, describe his method of using rubber plates in connection with piano-wire springs, I have used them more than ever before, and with increasing favor. There are many cases of irregularity where a rubber plate and a spring of piano-wire are all that is needed to accomplish the desired correction.

I have frequently corrected difficult and complicated cases with a single rubber plate to which had been attached springs and screws, some at the time the plate was made, others subsequently, as the malposed teeth had moved into their desired position.

The rubber plate possesses two desirable features,—namely, a

FIG. 1.

*A* represents the masticating surface of the movable shoe, showing gold facing.

*D* represents the inner surface of the fixed shoe.

*C*, the screw which fits into the screw cylinder *D*.

*E* shows the buttons to which the india-rubber bands are attached.

minimum amount of skill for its construction as well as a minimum expense, which is often a serious matter when precious metals are used and a number of appliances required.

To-day I inserted a little fixture for the purpose of pushing into the arch a lateral incisor which was within the line of the other teeth. There was room for it between the central and cuspid, but it was held in its abnormal position by the occlusion of the inferior lateral. A rubber plate was made capping the molars. That portion of the plate fitting against the palatine surface of the incisors was left quite thick, and opposite the malposed lateral a screw was embedded in the rubber. The screw was headless, and the slotted end of the screw rested upon the palatine surface of the lateral. The patient was given a small screw-driver, and requested to back that screw out a turn or half a turn twice or thrice daily, and then force the plate into position. As the plate bound the molars tightly, there was no slipping from its position when once forced into place by the lower teeth. If the girl is faithful in her use of the screw-driver the tooth will be in line in two or three weeks.

Such an appliance need not cost over two or three dollars, and the patient corrects the irregularity.

Rubber plates are a little cumbersome, and they are in many cases unclean, but this is the fault of the patient.

While I would not recommend them in all cases, I would not like to discontinue their use, nor have them condemned without a hearing.

Dr. Jack submitted a plate that he generally uses for carrying teeth out or drawing them inward. The plate was passed around among the members, and after illustrating the points upon which he wished to make his remarks by a black-board drawing, he said,—

"I want to call your attention to a form of plate I have been using for a great many years for the general purpose of aligning irregular front teeth and for forcing outward or inward the eight front teeth of either arch.

"In reference to the alignment of teeth, carrying them in when they are too far out and bringing them out when they are too far in, I use this plate almost invariably. It is composed of two pieces of vulcanite joined by a band of gold. (See Fig. 1.)

"The posterior teeth are made the base of resistance by covering the second bicuspid and the molars of both sides by two separate shoes of vulcanite, which extend at either side of the teeth but a few lines beyond the margin of the gum. To give these shoes strength and to enable the patients to masticate upon them, they are surfaced with gold swaged to the form of the ends of the teeth. These gold facings are vulcanized to the shoes in their proper places.

"Some preliminary preparation of the cast is required to enable these shoes to hold firmly their position. They should go on with a little springiness. The cast is trimmed with a suitable instrument to take a shaving from the teeth at the neck, and also a shallow groove should in most instances be made in the plaster, at the gingival margin. The proper amount of cutting is quickly gained by experience.

"These bases of support for the movement of the teeth are connected by a narrow band of springy gold, one end of the bar being secured to one of the shoes, the other end being attached to the opposite shoe by a male screw fitting in a screw-cut tube or, with proper precautions, vulcanized into a projection on the outer plate of the shoe.

"The reason for this plan is that by turning the free end of the appliance the bar may be reduced or increased in length. If in any given arch a tooth or more is projecting and others are depressed, the bar is brought into contact with the most prominent tooth, and a piece of elastic rubber is placed between this point of contact, at the same time a rubber ring is carried over each of the teeth which are within the arch and is drawn through a hole opposite the tooth and extended to a button. On the next day the bar is screwed up enough to be again in contact, when a new pressure may be made or the tooth is rested, as the conditions require. If the depressed teeth are sore, they may be rested by tying through the same channel as the ring had passed. I remove these plates daily, each time making a gain in the progress. It is important to make this daily change for the sake of cleanliness, the patient brushing the teeth while the further preparations are being made.

"As before intimated, all the front teeth may be moved at the same time either outward or inward.

"Rotation may also be conducted by the various attachments made for that purpose by connecting the rubber band to the attachment, and many modifications of this simple description will occur to meet the exigencies connected with the alignment of the teeth.

"It is almost needless to state that the impression of the teeth should be taken with plaster.

"This method may be made useful in the treatment of cases at a distance whenever either of the parents of the child has the intelligence to comprehend the mode of operation of the plate and is capable of applying the required instruction. In this connection I have conducted the correction of a great protrusion of the upper teeth and concurrent depression of the lower arch for a patient living a thousand miles from me, the mother each day making the necessary changes of the plate or ligatures. The upper teeth were forced backward in the manner described, and when their position was corrected, a similar plate was placed on the lower teeth, when they were gradually brought outward into correct relation with the upper arch. This necessitated but three periods of attention on my part.

"The only originality in connection with this appliance is the division of the old form of upper plate which was used to separate interlocked arches and to connect these by the screw at one end of the bar. I present this method to you as comprising many advantages for the purpose for which it is intended, and in this purpose is included the greater number of irregularities we have to treat.

"For the rotation of teeth and for bodily moving them on the line of the arch, my opinion is that no means are so effectual as the attachment of a screw connected with fixtures which are cemented to the base of resistance and to the irregularly-placed tooth.

"In reference to retaining fixtures, I employ the methods Dr. Guilford has mentioned to you. I invest the teeth with a fixture which is cemented firmly in place, sometimes covering the teeth entirely, at other times leaving a portion open. Sometimes I make them very extensive, covering the incisors, cuspids, and bicuspids. These are cemented on with oxyphosphate of zinc, and are worn from six to nine months."

This closed the discussion of the subject.

Dr. Deane, under "Incidents of Practice," asked the advice of the members as to how to proceed in a case he had, where a boy, about thirteen years of age, had fallen against a curb-stone and broken off the right central about three-fourths of its length; the opening at the apex of the root was quite large. Would it be better to restore the crown? The root is in good condition, except that the apex of the root is not closed. Should it be extracted or left as it is?

Dr. Truman suggested the use of oxychloride of zinc.

Dr. Darby stated that in a somewhat similar case he made a little piece of gold to go into the canal, with a thread cut upon it. He suggested mounting the crown.

Dr. Gaskill stated a case where a tooth had been broken off and subsequently gave the person trouble. Upon examining the tooth he found an opening an eighth of an inch in diameter. A piece of tooth-brush handle was taken and made to fit the cavity exactly, and coated with chlora-percha, inserted in the root, and a crown mounted. It had served the purpose very well, with no subsequent trouble.

Dr. Boice stated that he had a case a number of years ago in which the apex was larger than any part of the canal. In drilling it out he had to hold the tooth. Finally, it was filled with cotton, expecting the patient to come back in a few months, but it was sixteen or seventeen years ago and had given satisfaction all that time.

Dr. Jack stated that he had the history of four such cases. He measured the exact distance to the end of the root with a broach; then fitted a steel probe by filing it to fit exactly the apical opening, and cut it off at a point where it would not go through; this gives the exact size of the opening; he then prepares a cylinder of gutta-percha on a warmed porcelain plate to the proper size; then cuts a section of the cylinder at a point a little larger than the piece of steel. This is next attached to the probe, carried up and forced by measure to the proper place, the gutta-percha being obliged to adapt itself to the form of the canal at the apex. Afterwards the remainder of the root is carefully filled.

Dr. Guilford called the attention of the members to the adaptability of platinized silver for dowels, being less expensive than gold, and standing any amount of heat any article can be subjected to except platinum. The doctor stated that he frequently soldered it with 20-carat gold. It has not quite the stiffness of platinized gold, but where less stiffness is required it answers the purpose perfectly well. It can be bought of Ash & Son, New York.

Dr. Bonwill then read a proposition for the consideration of the Society in regard to the proper disposition of patented articles.

After Dr. Bonwill had read his paper, the consideration of his proposition was taken up and opened by Dr. Boice, who said that he understood Dr. Bonwill had something which he wanted to present to the Society; that he had given a good lecture on patents but was not able to see that the doctor had given the Society anything. Dr. Boice further said that he did not feel that he could offer any motion accepting anything that had not been offered.

Dr. Bonwill answered that if there were no clause in it upon which they could take action they might accept it and place it before the American Dental Protective Association.

Dr. McQuillen stated that, as he understood it, Dr. Bonwill wanted the Society to go on record as favoring patents of all kinds, and to present this paper with its endorsement.

*Dr. Truman.*—Dr. Bonwill desires that this Society shall refer this matter to the American Dental Protective Association. In doing that, as I apprehend it, we do not endorse it.

Dr. Darby moved to place it before the American Dental Protective Association, with a disclaimer of any endorsement.

This motion was carried.

## Figures and Tables

**Fig. 1. f1:**